# Model-Based Tracking of Fruit Flies in Free Flight

**DOI:** 10.3390/insects13111018

**Published:** 2022-11-03

**Authors:** Omri Ben-Dov, Tsevi Beatus

**Affiliations:** 1 The Institute of Life Science, The Hebrew University of Jerusalem, Jerusalem 9190401, Israel; 2 School of Computer Science and Engineering, The Hebrew University of Jerusalem, Jerusalem 9190401, Israel; 3 Center of Bioengineering, The Hebrew University of Jerusalem, Jerusalem 9190401, Israel

**Keywords:** insect flight, tracking, pose estimation, drosophila

## Abstract

**Simple Summary:**

One of the main bottlenecks in studying the flight of insects is automatically measuring the motion of their body and wings. Here, we report a computer vision algorithm for this task based on three fast-camera views and a 3D model of the insect. We demonstrate the performance of this method on the free and maneuvering flight of fruit flies. This method is potentially applicable to other insect species.

**Abstract:**

Insect flight is a complex interdisciplinary phenomenon. Understanding its multiple aspects, such as flight control, sensory integration, physiology and genetics, often requires the analysis of large amounts of free flight kinematic data. Yet, one of the main bottlenecks in this field is automatically and accurately extracting such data from multi-view videos. Here, we present a model-based method for the pose estimation of free-flying fruit flies from multi-view high-speed videos. To obtain a faithful representation of the fly with minimum free parameters, our method uses a 3D model that includes two new aspects of wing deformation: A non-fixed wing hinge and a twisting wing surface. The method is demonstrated for free and perturbed flight. Our method does not use prior assumptions on the kinematics apart from the continuity of the wing pitch angle. Hence, this method can be readily adjusted for other insect species.

## 1. Introduction

Insect flight is an impressive example of highly maneuverable and robust locomotion [1,2]. It both challenges our scientific understanding and inspires us to develop tiny bio-mimetic drones [3]. The mechanisms underlying many aspects of insect flight such as control, navigation, aerodynamics and sensory integration, as well as their neural and genetic basis, are subjects of active research. Modern high-speed cameras and computational tools have greatly advanced insect flight research. Yet, a significant bottleneck in this field is automatically extracting accurate kinematics from large amounts of multi-view free-flight videos, where the main challenges are wing deformations and occlusions. Here, we refer to this data extraction process as “tracking”.

Current tracking methods can be divided into several categories. (1) *Manual tracking*, where a 3D model of the insect is manually fitted to individual frames, is relatively accurate but extremely laborious [4,5,6]. (2) *Landmarks tracking* of feature points on the insect body and wings [7,8,9]. This method might require gluing markers on the insect wings, might suffer from marker occlusion, and often requires manual input. (3) *Structured light illumination* has been used to track dragonfly wings and their deformation but is currently limited to large insects [10]. (4) *Hull reconstruction* methods generate a 3D hull of the insect by tracing the rays from each pixel in each camera view. The hull is segmented into body and wings voxels, from which the insect degrees of freedom (DOFs) are extracted [11,12,13,14,15]. (5) *Model-based* methods fit a 3D insect model by projecting it onto the camera planes and matching the projections to the data images [16,17] or by fitting the model to a 3D hull [18]. This approach, first applied for flies in [16], was used in later works (e.g., [19]) for analyzing many flight events. Obtaining accurate results using this approach requires a 3D model that mimics the insect and its DOFs very faithfully. For example, insect wings are typically not rigid and deform during flight [20], and the wing hinge, connecting the wing to the insect body, is flexible. These deformations cannot be described by modeling the wing as a rigid plate connected at a fixed hinge point.

In this paper, we present a model-based algorithm for extracting free-flight kinematics from high-speed multi-view videos of fruit flies. Our 3D model embodies realistic wing deformations and moving wing roots using only a few additional parameters. Motion DOFs are obtained by minimizing an intuitive loss function, and the reported results include no filtering or smoothing. This method may alleviate a significant data analysis bottleneck, thereby allowing us to analyze complex phenomena, such as flight control and sensory integration, with high statistical power.

## 2. Materials and Methods

### 2.1. Problem Definition

**Figure 1:**
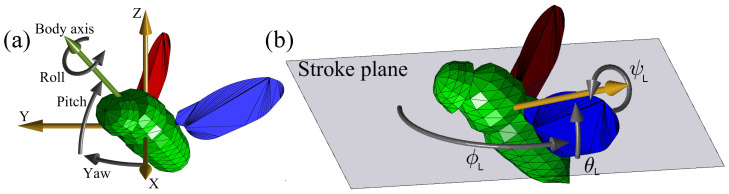
Basic 12-DOF model parameters. (**a**) Body 6 DOF describing its position and orientation. (**b**) Each wing is described by 3 Euler angles relative to the stroke plane: Stroke (ϕ), elevation (θ) and wing pitch (ψ). The annotations are for the left wing.

We aim to solve the pose estimation problem for fruit flies (*Drosophila melanogaster*) in free flight. The input consists of multi-view videos of a fly, and the output is its body and wing kinematics. Body parameters (Figure 1a) consist of 6 DOFs: 3 translational DOFs (x,y,z coordinates) and 3 Euler angles (roll, pitch, yaw). The wing parameters are Euler angles that represent wing rotation (Figure 1b): the stroke angles $\phi_{\ell },\phi_{r}$ represent the wing’s forward and backward sweeping motion within the stroke plane; the elevation angles $\theta_{\ell },\theta_{r}$ describe wing elevation with respect to the stroke plane; and the wing–pitch angles $\psi_{\ell },\psi_{r}$ measure wing rotation around its leading edge. Thus, the basic kinematic description of the fly consists of 12 DOFs.

### 2.2. Experimental Setup

**Figure 2:**
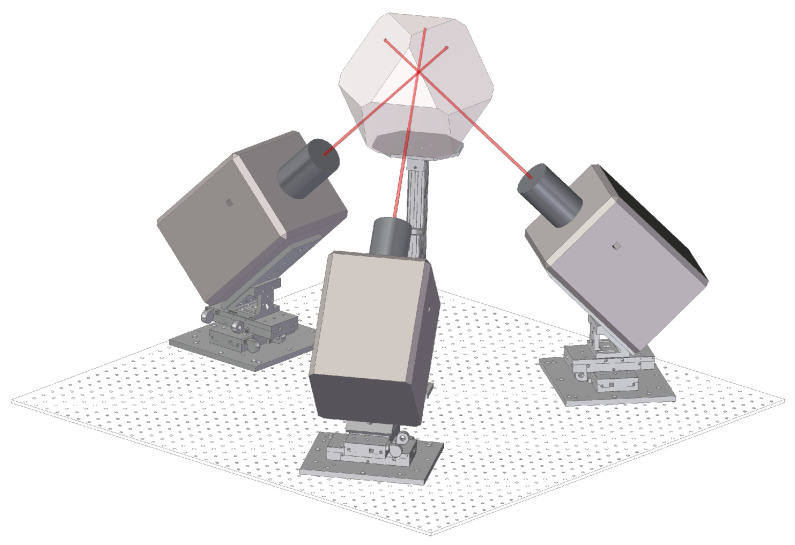
Experimental setup. Three orthogonal high-speed cameras focused on a transparent chamber. The non-Cartesian setup reduces wing occlusions.

The experimental setup (Figure 2) consists of 3 orthogonal high-speed cameras (Phantom v2012, Vision Research, Wayne, NJ, USA), operating at a rate of up to 22,000 frames/s and 1280×800 pixel resolution. The cameras are back-lit by IR LEDs and tilted upwards by $∼36^{{}^{\circ}}$ to reduce wing–wing and body–wing occlusions with respect to a Cartesian camera configuration, which aligns with the lab frame of reference. The volume mutually seen by the cameras is $∼5\;\times \;5\;\times \;5\;{\mathrm{cm}}^{3}$, which is located at the center of a custom-made 3D-printed cage. The camera system is calibrated [21], allowing us to convert between 3D world-points and 2D image-points. In total, 10–30 female *D. melanogaster* flies (Canton S line, 2–5 days old) were placed in the cage and recorded as they flew through the filming volume. To study insect flight control, we exerted mechanical perturbations to the flies by gluing a tiny magnet to the back of each fly and using a magnetic pulse to rotate it in mid-air [13,14,22].

### 2.3. Background Subtraction

**Figure 3:**
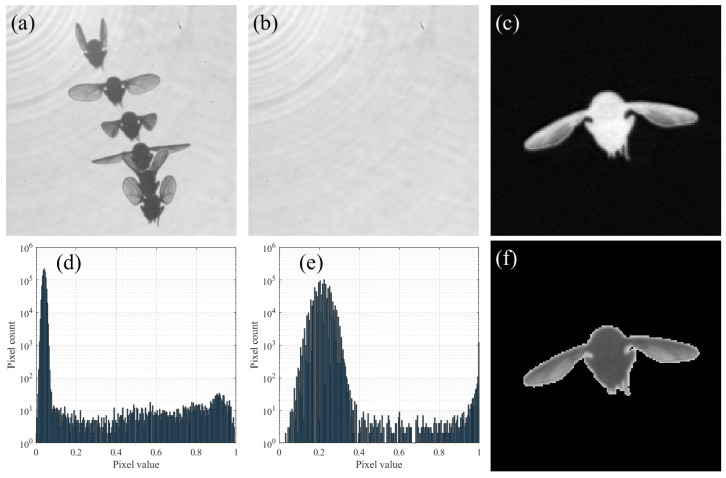
Pre-processing of free flying videos. (**a**) Superimposition of 6 raw images mid flight. (**b**) Background extracted by taking pixel-wise maximum. (**c**) Subtraction of a single frame from the background. (**d**) Histogram of subtracted image. The distribution is almost uni-modal. (**e**) Histogram of subtracted image after power transformation. Distribution is more bi-modal. (**f**) Resulting mask after applying Otsu’s threshold on transformed difference image.

Back-lighting makes the fly pixels darker than the background (Figure 3a). Thus, the background is computed by taking the pixel-wise maximum across two frames: the first and last frames in the video (Figure 3b). This method avoids any overlap of the fly with itself, relying on the verified assumption that the videos are long enough such that the first and last frame show the fly in different positions. To obtain a binary mask from each frame, we first subtract its background, and we use the transformation $p\to 1-{\left(1-p\right)}^{6}$ on each pixel value *p* (Figure 3c). This transformation makes the wings relatively brighter such that the pixel-value distribution becomes closer to bimodal (Figure 3d,e). Then, we apply Otsu’s binary threshold method [23], which relies on this bi-modality, and obtain the fly’s mask (Figure 3f).

### 2.4. Generative Model

Our model consists of geometric 3D descriptions of the fly’s body and wing. The model for the fly’s body is based on [16] with some rescaling and a modified head pose. Additionally, to analyze perturbation videos, where the fly had a magnet glued on its back and was rotated by an external magnetic field, we included the magnetic rod in the 3D model of the body. The wing model was obtained by imaging a fly’s wing on a microscope and tracing its outline.

**Figure 4:**
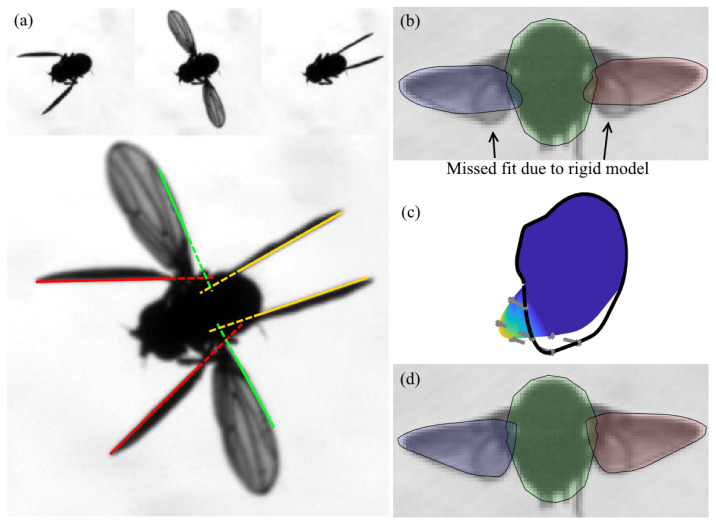
Wing deformations. (**a**) Top: Three frames from different phases of a single wing beat. Bottom: Superimposing the 3 frames shows that the wing hinge is effectively not fixed during the stroke. The solid lines marking the leading edge of the wing do not intersect at a single point (dashed lines). (**b**) An unsuccessful fitting attempt using a rigid wing on a frame with a twisted wing during supination. (**c**) Wing deformation used in our 3D model. Color represents deformation level, and the black line shows the rigid wing outline. (**d**) A successful fit using a flexible wing.

The accuracy of model-based pose estimation strongly depends on how well the model and its DOFs mimic the target object. We found that using the 12 DOF description (Figure 1) leads to significant tracking inaccuracies, because this model does not include two important geometric features of the fly (Figure 4). First, due to the flexibility of the wing base, the wing hinge cannot be accurately described as a single point (Figure 4a). In our model, this feature is described by allowing the two wing hinges to translate symmetrically with respect to the body, which requires 3 additional kinematic parameters: δx, δy and δz, which represent hinge translation in the body frame of reference. Allowing asymmetric hinge translation (3 translational DOFs for each wing hinge) hindered the optimization process, because it favored the motion of the wing hinges over the motion of the wing angles.

Second, the wing surface deforms due to the interplay between aerodynamic, inertial and elastic forces acting on the wing [12,20,24,25,26,27]. Although these deformations are small, compared with wing deformation in other insects [28], they cannot be captured by a rigid wing model, which introduces sizeable tracking errors especially during wing pronation and supination (Figure 4b). In our model, wing deformation is described by a single parameter per wing: $\alpha_{\ell },\alpha_{r}$. As observed experimentally, wing deformation is largest near its base and decreases toward the wing tip [20,25]. Each α parameter quantifies twist per unit length; twist increases linearly from the wing tip (no twist) to the wing base (maximum twist). The model wing is twisted only at the bottom half below its center-line (Figure 4c).

Overall, our model consists of 17 kinematic parameters: the standard 12 DOFs, 3 symmetric translational offsets of the wing hinges, and 2 twist parameters (Table 1).

### 2.5. Loss Function Optimization

To quantify the disagreement between the model and a single image, we first project the 3D model onto the corresponding camera plane. The projection of the model’s 3D vertices is calculated according to the pinhole camera model. Each vertex $v=\left(x,y,z\right)$ of the model is projected to a 2D pixel location $p=\left(i,j\right)$ using the camera matrix *M* obtained from our calibration procedure:

(1) $$\left(\begin{array}{c} i^{\prime }\\ j^{\prime }\\ t \end{array}\right)=M\left(\begin{array}{c} x\\ y\\ z\\ 1 \end{array}\right);\;i=\frac{i^{\prime }}{t},\;j=\frac{j^{\prime }}{t}$$

**Figure 5:**
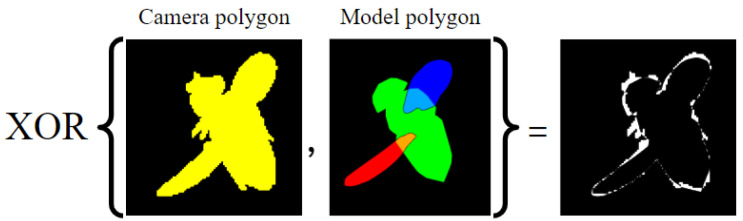
Single-frame loss function. XOR operation on the camera image mask (yellow) and the projected model.

To compute a 2D polygon per camera, we apply Graham’s scan over the resulting 2D vertices. However, since Graham’s scan returns convex hulls, we first compute a polygon for the body and each wing separately. We then compute the full model polygon as the union of these three polygons. The single-view loss function is defined as the non-overlapping area (*XOR*) between the model polygon and fly’s binary mask (Figure 5). The *XOR* operation is equivalent to the subtraction of the intersection of the two images from their union. To compensate for the different apparent sizes of the fly in each view, we normalize each *XOR*-ed area by the area of the corresponding mask:

(2) $$L\left(p\right)=\frac{\mathrm{Area}\left[{\mathrm{mask}}_{\mathrm{fly}}⊕\mathrm{model}\left(p\right)\right]}{\mathrm{Area}\left[{\mathrm{mask}}_{\mathrm{fly}}\right]},$$

where p is the parameters vector, ${\mathrm{mask}}_{\mathrm{fly}}$ is the fly’s binary mask and $\mathrm{model}\left(p\right)$ is the polygon of the 3D model’s projection.

The multi-view loss function is a weighted mean of the single-view losses. As tracking the wings is more difficult than tracking the body, we assign greater weight to views that hold more information about the wing pose. The weight of each view, calculated from the initial condition, is proportional to the percentage of wing area unoccluded by the body.

To evaluate the model parameters at a given time-point, we minimize the multi-view loss function using a derivative-free interior-point method (*fmincon* in Matlab). Prior to the optimization process, all parameters are scaled to the range $\left[0,1\right]$ to balance the relative weight of the loss in each parameter. After the optimization, the results are scaled back to the original ranges. The initial condition for the optimization is the result of the previous frame. The initial condition for the first frame is obtained semi-automatically, where the user applies manual adjustments to automatic optimization results via a graphical user interface. At this step, the user can also determine constant scaling parameters of the model to handle flies of different sizes. Each fitted angle was constrained to its physiologically possible range known for flies. The body roll angle was constrained to a range of $\pm 2^{{}^{\circ}}$ with respect to the initial condition for each frame.

**Figure 6:**
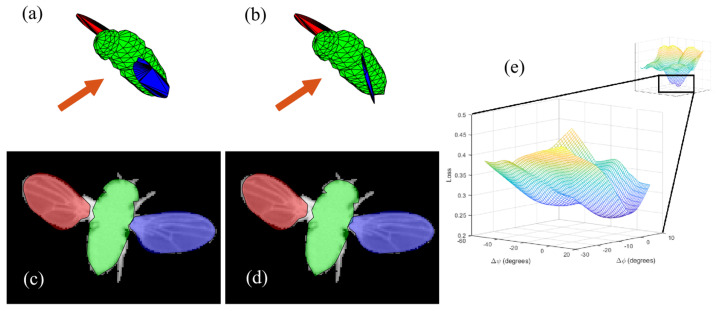
Degeneracy in ψ. (**a**,**b**) The 3D model generated by two sets of parameters. The orange arrow shows the direction of the camera taking the images on the bottom. (**c**,**d**) The projection of the corresponding models on the camera plane. The projections are nearly identical. (**e**) The loss function (*z*-axis) at the presented frame by changing only $\psi_{\ell }$ and $\phi_{\ell }$ (*x*-axis and *y*-axis, respectively).

We identified that the combination of our loss function and camera configuration leads to degeneracy of the model in certain body and wing poses. As shown in Figure 6, two values of the wing pitch angle ψ of the left wing, which differ by $∼30^{{}^{\circ}}$, generate almost identical projections of the model. This degeneracy is observed almost exclusively in the wing pitch angle, ψ. Consequently, in such cases, optimization might converge to a wrong local minimum (Figure 6e). To address this degeneracy, we apply an error detection and correction protocol, which exploits temporal information by detecting discontinuities in either ψ or the loss function. Then, in the correction step, we use a multi random start (MRS) procedure, in which we restart the optimization process from 15 random points in parameter space and then re-fit previous ‘suspected’ frames using the same constraints as detailed above. In order to increase the chance of finding the best minimum, the sampled random points are spread over the sampling volume using *k-means++* seeding [29]. In this method, 10,000 points are first randomly sampled uniformly from the sampling volume. Of these points, a single point is chosen randomly with uniform distribution. Then, for 14 iterations, a new point is randomly chosen using a distribution that is proportional to the distance from the nearest already chosen point. This process results in points which have a high probability to be distant from each other, thus spreading over the sampling volume.

## 3. Results

### 3.1. Validation

**Figure 7:**
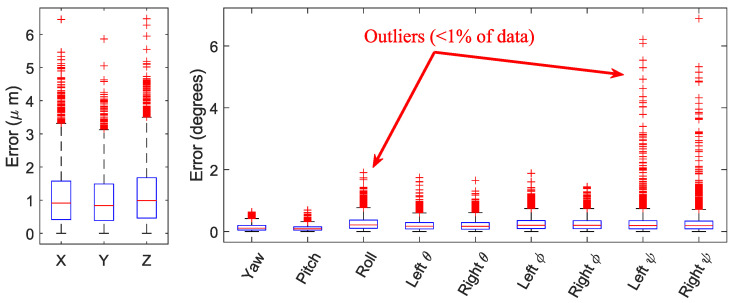
Model validation on synthetic data. Tracking errors box plot. Each box contains 75% of the data. Whiskers correspond to 99.3% of the data.

To validate our method, we tested it on an ensemble of synthetic images generated from the basic 12-DOF model used for optimization. We used previously measured and manually corrected flight kinematics [13] to generate 36 videos of 100 time points each (a single wing beat). Each video differs by the body yaw angle. Figure 7 shows a box plot of the resulting errors for each DOF. The fly’s center of mass position was accurate within 10μm (≈0.2 pixel). In the angular parameters, in 98% of the frames, the error in all angles was less than $2^{{}^{\circ}}$.

**Figure 8:**
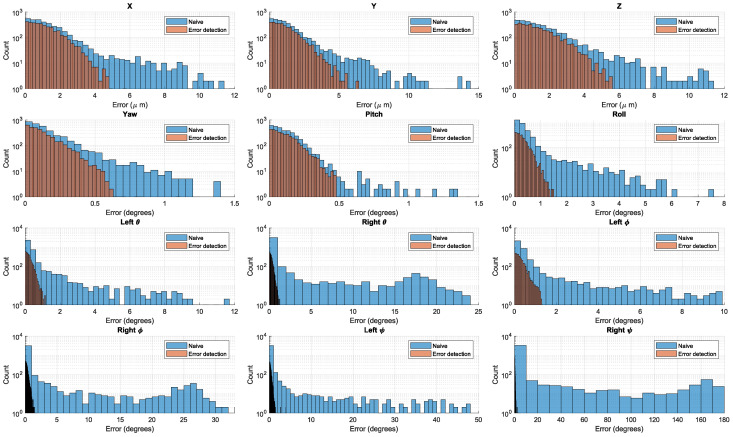
Naive errors vs. error detection. Each graph is a histogram of the errors for each DOF. The blue bars are the naive optimization process and the orange bars are the process using error detection.

To demonstrate the effect of error detection and correction, we ran the same process again (on the same videos) with a naive optimization: switching off error detection. The histogram of the errors (Figure 8 shows the marked improvement made by the error detection and the MRS error correction steps, particularly for the wing angles.

For example, the errors of the naive algorithm in $\psi_{r}$ (bottom right plot) reached $180^{{}^{\circ}}$, which is a state where the model wing is upside-down with respect to the ground truth. In contrast, when using error detection and correction, 99.75% of the errors in $\psi_{r}$ were less than $2^{{}^{\circ}}$.

### 3.2. Non-Maneuvering Flight

**Figure 9:**
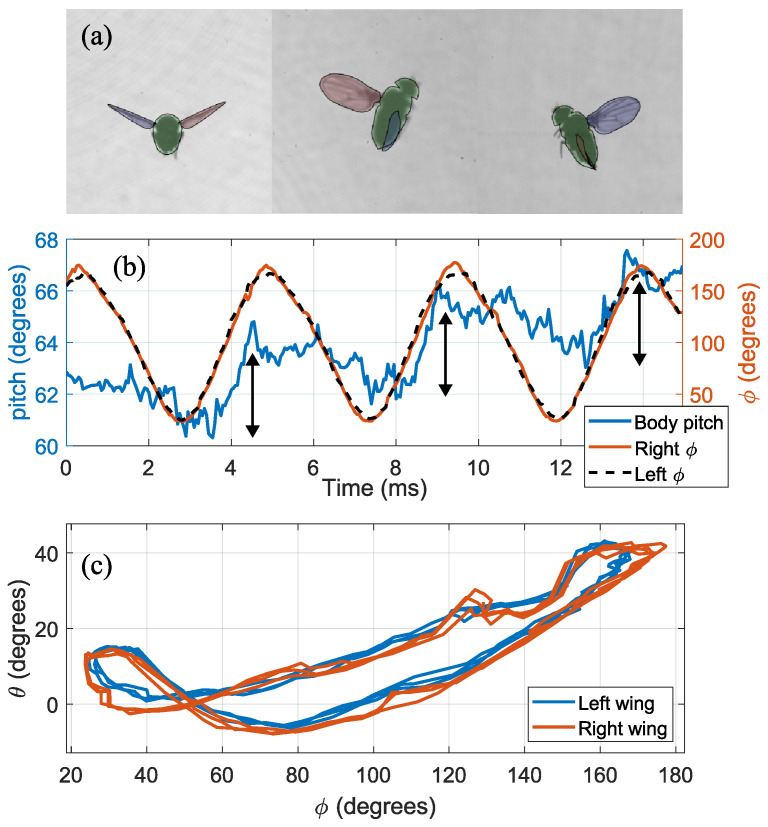
Results on an unperturbed flight event. (**a**) The projection of a fitted 3D model superimposed on the corresponding frames. (**b**) Body pitch and wing ϕ. Body pitch oscillations are marked in black vertical arrows. (**c**) The path of the wing tip by its elevation (θ) and azimuth (ϕ).

**Figure 10:**
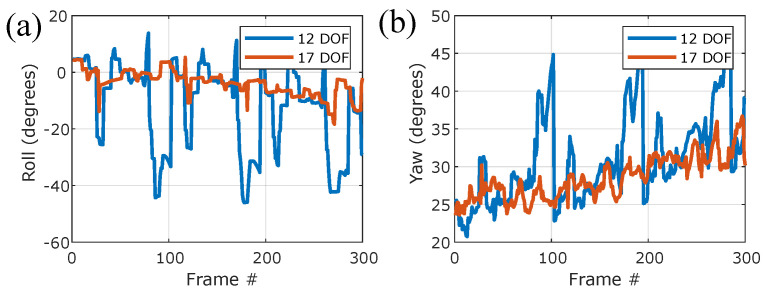
Comparison between the 12-DOF and 17-DOF models. The body roll (**a**) and yaw (**b**) angles found using the 12-DOF (blue) and 17-DOF (red) models, both for the same unperturbed, non-maneuvering flight data.

Figure 9 and Supplementary Video S1 demonstrate the pose estimation of an experimentally measured free-flight sequence with no maneuvers. Interestingly, the oscillations in the body pitch angle (Figure 9b) correspond to the natural periodic pitch motion of the fly: when the wings are in the forward half of the stroke plane (ϕ<90), they exert a pitch-up torque on the body, and when ϕ>90, the wings exert a pitch-down torque [14]. Together, these torques result in small, $∼2^{{}^{\circ}}$ amplitude pitch oscillations that are clearly seen in both the raw videos and measured data. Tracking the wing angles (Figure 9c) shows the typical figure-8-like trajectory of the wing tip. Comparing the 17-DOF model to the rigid 12-DOF model (Supplementary Video S2), we find that for the 17-DOF model, the mean loss across the entire video was 0.1049±0.0068 (mean ± standard deviation), which is better than the loss of fitting the rigid 12-DOF model, which was 0.1501±0.0204. Figure 10 compares the results of the 12-DOF and 17-DOF models on the same sequence of frames. The 12-DOF model shows much larger errors, which are temporally aligned with times when the wing twists the most.

### 3.3. Free Flight Maneuver

**Figure 11:**
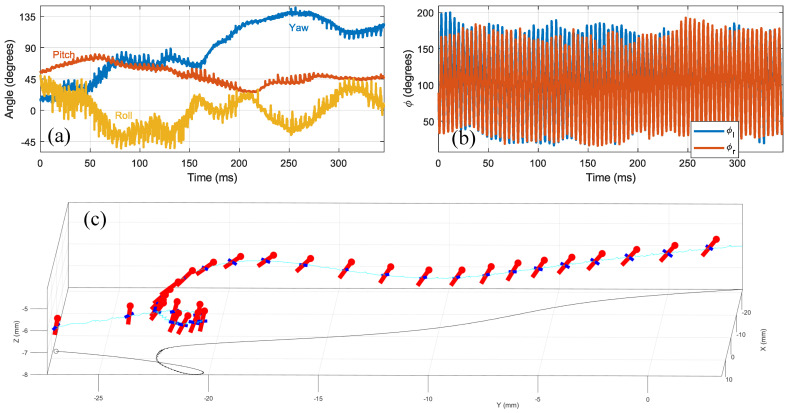
Free flight maneuver. (**a**) Body angles. (**b**) Wings stroke angles. (**c**) A drawing of the path and body angles of the fly. The red rods represent the orientation of body at different time points, with the circular end marking the head. The blue rods attached to the red rods represent the wing span vector, visualizing the yaw and roll angles. A small circle on the left marks the start of the video.

Figure 11 and Supplementary Video S3 show the pose estimation results for a free flight maneuver in which the fly performed a horizontal loop and then accelerated forward (5527 frames, 345ms). The algorithm captures the variations in the body position and orientation (Figure 11a) as well as the modulations in the wing kinematics (i.e., the stoke angle Figure 11b) which are associated with the observed maneuver.

### 3.4. Roll Correction Maneuver

**Figure 12:**
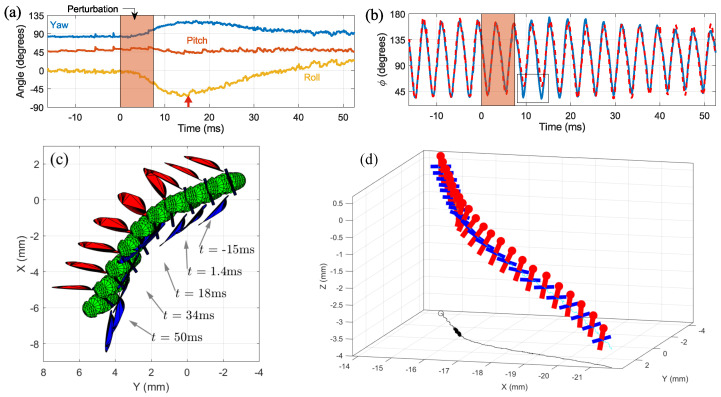
Roll correction. (**a**) Body angles during the maneuver. Magnetic pulse was activated between t=0−7.5ms. An orange vertical arrow marks the time of maximum angular deflection in roll, yaw and pitch. (**b**) Wings stroke angles. Blue line and red dashed line mark $\phi_{\ell }$ and $\phi_{r}$ respectively. The rectangle marks the main wing asymmetry during the maneuver. (**c**) Top view of the fitted model shows every two wing beats when the left wing is at supination. Wing stroke asymmetry is clearly visible. (**d**) Flight trajectory and body pose plotted in 1 wingbeat intervals during the maneuver. The body *x* axis is plotted in red lines, with a red dot indicating the head, and the body *y* direction is represented by blue line. The trajectory starts at the top corner of the plotted box. A 2D projection of the trajectory is plotted on the xy plane (black line), with the time of the perturbation marked by a thicker line.

Figure 12 and Supplementary Video S4 show the pose estimation of a measured roll correction maneuver in response to a mid-air magnetic perturbation (Section 2.2). Here, we modified the 3D model to include the magnetic rod and determined its position manually along with the initial condition. Tracking the body angles (Figure 12a) shows that the fly was rolled to its left by $62^{{}^{\circ}}$ at t=15ms after the onset of the perturbation. Body yaw and pitch were also perturbed by $-12^{{}^{\circ}}$ and $40^{{}^{\circ}}$, respectively, because the magnetic torque is typically not aligned with any body principal axis. Tracking the wing stroke angles demonstrates the fly’s roll control mechanism [13], where the ‘bottom’ wing (here, left) increases its stroke amplitude and the ‘top’ wing decreases its stroke amplitude. The roll reflex latency was ≈9ms and the perturbation was fully corrected after ∼9 wing beats (t≈40ms). A characteristic feature of these maneuvers is a residual error in the body yaw angle with respect to its value before the perturbation [13]. In this example, the fly’s yaw error was $10^{{}^{\circ}}$.

## 4. Discussion and Conclusions

We presented a pose-estimation algorithm for tracking free-flying fruit flies. The novel features of the model include wing deformation, non-fixed wing-hinge and the addition of a magnetic rod for perturbation experiments. Importantly, the results shown here did not undergo any smoothing or filtering. Furthermore, our algorithm does not use any prior assumptions on the kinematics, except for the continuity in ψ for error detection. Overall, the results of this algorithm are less noisy than the results of the hull reconstruction algorithm reported in [13,14], which is, in turn, based on [11] (Appendix A). This comparison also shows that the current algorithm is robustly applicable, without any changes, for analyzing fruit fly data taken in a different experimental setup, with different camera configurations, frame rates and resolution. Applying this approach to other insects would require providing a parameterized 3D model of the insect and calibrating the camera setup. Future improvement of this method may include (1) modeling, or at least removing, the insect’s legs, which might introduce tracking errors; (2) improving the accuracy in estimating the wing pitch angle, which is the most difficult DOF to measure, possibly by exploiting additional information from the grayscale images; (3) fully automating the process of finding the initial condition for the first frame; (4) using additional camera views; and (5) using differential rendering [30], which will allow us to utilize gradient-dependent optimization algorithms. Overall, this work defines a streamlined data analysis pipeline that can be easily converted to work with other types of insects.

## Figures and Tables

**Table 1:** Definition of model degrees of freedom.

Name	Units	Description
x,y,z	mm	Center of mass position in the lab frame
yaw	deg	Body azimuth angle (rotation around lab *z*)
pitch	deg	Body elevation angle
roll	deg	Body rotation around body *x* axis
$\phi_{\ell },\phi_{r}$	deg	Wing stoke angles
$\theta_{\ell },\theta_{r}$	deg	Wing elevation angles
$\psi_{\ell },\psi_{r}$	deg	Wing pitch angles
δx,δy,δz	mm	Wing hinges translation in the body frame
$\alpha_{\ell },\alpha_{r}$	deg/mm	Wing twist per mm

**Figure A1:**
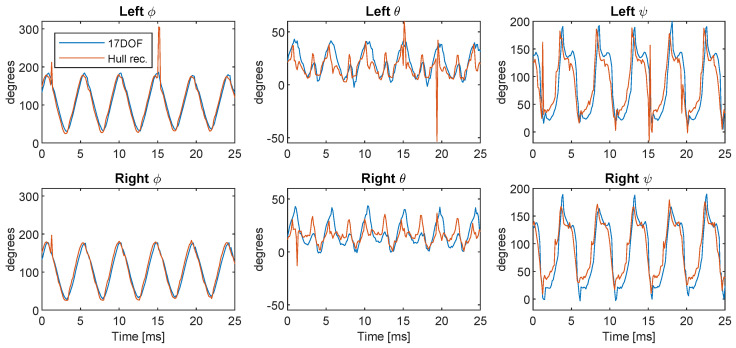
Comparing the 17-DOF model to hull reconstruction tracking in a Cartesian camera configuration. The six plots show 25 ms of wing angles data for both wings, comparing the results of two algorithms: The 17-DOF fitted model reported here (blue) and the hull reconstruction tracking reported in [11,13,14] (red). The video was taken at 8000 frames per second and 512×512 pixel resolution.

**Figure A2:**
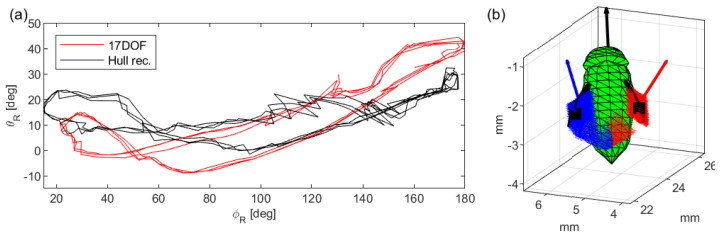
Comparing the 17-DOF model to hull reconstruction tracking in the current camera configuration. The unperturbed flight video from Figure 9 was analyzed using a hull reconstruction algorithm which approximates the performance of the methods reported in [13,14] by adjusting the latter method to the current camera configuration, higher resolution and higher frame rate. (**a**) The elevation angle of the right wing $\theta_{R}$ as a function of the stroke angle of the same wing $\phi_{R}$. Results of the 17-DOF model (red) are less noisy and more self-consistent than the results of the hull reconstruction method, especially when the wings are at the back ($\phi_{R}=130-180^{{}^{\circ}}$). (**b**) The reconstructed hull and the fitted 17-DOF model, both superimposed. In this video frame, when the wings are at the back, wing–wing and body–wing occlusions result in a oversized wing hull, which causes errors in the wing pose estimation. For example, both chord vectors are wrongly identified by the hull reconstruction method (red and blue line). In this example, the wings’ DOFs are correctly identified by the 17-DOF model.

## Data Availability

The data presented in this study are available on request from the corresponding author.

## Supplementary Materials

The following supporting information can be downloaded at: https://www.mdpi.com/article/10.3390/insects13111018/s1, Videos S1–S4: Video S1 (unperturbed flight 17 DOF model) shows the 17-DOF model superimposed on a short flight sequence. Video S2 (unperturbed flight 12 DOF model) shows the 12-DOF model superimposed on a short flight sequence. Video S3 (free flight manuever 17 DOF model) shows the 17-DOF model superimposed on a longer flight sequence and the complete path computed by the model. Video S4 (roll correction maneuver) shows the 17-DOF model with magnet attached superimposed on a flight sequence with a roll perturbation.

## Abbreviations

The following abbreviations are used in this manuscript:

DOF	degree of freedom

## Appendix A. Comparison to 3-Camera Hull Reconstruction

To further evaluate the performance of our method, we compare its output to the results of the hull reconstruction motion tracking algorithm used in [13,14], which is, in turn, based on [11]. The results of both algorithms were compared without any smoothing or manual correction. First, we compare the wing angles extracted from a free-flight video that was taken using a different camera configuration than the one used here: three co-orthogonal cameras aligned with the lab frame of reference, as in [11]. The comparison in Figure A1 shows that the 17-DOF model is less noisy. Next, we compare the two methods on the unperturbed flight video from Figure 9, which was taken using the camera configuration used in this work (Figure 2). Figure A2a plots θ vs. ϕ of the right wing, similarly to Figure 9b. The comparison shows that the 17-DOF model (red line) is less noisy and more self-consistent, especially when the wings are at the back of the fly (ϕ approaching $180^{{}^{\circ}}$). Figure A2b highlights the main reason for this difference in performance: when the wings are at the back, wing–wing and wing–body occlusions result in larger wing hulls, which causes errors in the wing pose estimation.
